# Sleep quality during preparation for RAI: recombinant TSH vs. levothyroxine withdrawal

**DOI:** 10.3389/fendo.2025.1683115

**Published:** 2025-10-24

**Authors:** Georgios K. Markantes, Konstantinos Dimitropoulos, Anastasia Armeni, Niki Chrysanthopoulou, Konstantinos Georgiou, Irene Mamali, Neoklis A. Georgopoulos, Anastasia Theodoropoulou

**Affiliations:** Division of Endocrinology - Department of Internal Medicine, University of Patras School of Health Sciences, Patras, Greece

**Keywords:** thyroid cancer, radioactive iodine, preparation, sleep, recombinant human thyrotropin (rhTSH), levothyroxine withdrawal

## Abstract

**Background:**

Patients with differentiated thyroid cancer (DTC) suffer from impaired sleep quality, which may be further impaired after radioactive iodine (RAI) treatment. The potential contribution of the type of preparation for RAI has not been studied. Our objective was to compare the effect of the types of preparation for RAI on the sleep quality of DTC patients, and to correlate sleep indices with hormonal and psychological parameters.

**Methods:**

we studied 104 DTC patients (76 women and 28 men) programmed to receive RAI, two to eight months after total thyroidectomy. Participants were classified into two groups, based on the type of preparation: 56 were prepared with levothyroxine withdrawal (LT4-W group) and 48 with recombinant human TSH (rhTSH) administration (rhTSH group). Assessment was done in the morning of RAI administration and included hormonal determinations (thyroid function tests, prolactin, cortisol, and adrenocorticotropic hormone) and evaluation of sleep quality (Pittsburg Sleep Quality Index-PSQI) and anxiety/depression levels (Hospital Anxiety and Depression Scale-HADS).

**Results:**

The two study groups did not differ in age and gender distribution, DTC histology, RAI indications and administered dose, thyroglobulin and anti-thyroglobulin antibodies levels. The rhTSH group demonstrated higher TSH and free thyroxine, and lower prolactin and cortisol; participants of this subgroup had better subjective quality (0.65 ± 0.71 vs 1.22 ± 0.92, p=0.002) and efficiency (0.35 ± 0.74 vs 0.72 ± 0.96, p=0.023), less daytime dysfunction (0.52 ± 0.59 vs 0.87 ± 0.82, p=0.03), and better global PSQI score (4.72 ± 3.32 vs 6.60 ± 3.99, p=0.014), while they also showed lower anxiety/depression scores compared to individuals in the LT4-W group (4.32 ± 4.22 vs 5.62 ± 3.85, p=0.046 and 3.55 ± 3.41 vs 5.86 ± 4.22, p=0.005, respectively). The between-groups differences in the above PSQI domains remained after correction for hormonal levels but disappeared (except for that in subjective quality) after correcting for anxiety/depression levels.

**Conclusions:**

In DTC patients receiving RAI, preparation with rhTSH could contribute to better sleep quality compared to LT4 withdrawal. Increased depression and anxiety rather than differences in hormone levels seemed to mediate this difference.

## Introduction

Sleep is a naturally recurring, complicated process characterized by decreased consciousness, sensory and bodily activity that occupies approximately one third of the average human lifespan. Although the exact purpose of sleep is still poorly understood, its high importance for a wide range of physiological functions and overall health is well-established (1). The Ancient Greeks recognized the value of sleep and deified it, demonstrating how important it was for their heath and spirit. The endocrine system, via the secretion of hormones, also regulates a variety of bodily functions and maintains homeostasis. Thus, it is not surprising that sleep and the endocrine system demonstrate high levels of interdependence, both in health and in disease. Most hormones have a circadian rhythm, which is influenced by the sleep-wake cycle (1, 2).

Thyroid function in particular is significantly affected by sleep; night-time sleep attenuates the physiological nocturnal rise of thyrotropin (TSH) and thyroid hormones (TH: thyroxine-T4 and triiodothyronine-T3), while alterations in sleep duration and/or quality have composite effects on TSH and TH (3–5). In turn, thyroid dysfunction (both hyper- and hypothyroidism) has been consistently associated with sleep related disorders, such as impaired sleep quality and insomnia, hypersomnia, obstructive sleep apnea (OSA) and restless legs syndrome (1, 6, 7). Sleep disorders constitute a heterogeneous group of various conditions, which affect over a third of the adult population; gender distribution differs according to the specific condition: for example, insomnia is 1.5 times more common in women, while obstructive sleep apnea is 2 times more prevalent in men (8). Sleep is also adversely affected by psychiatric disorders, the most common of which are anxiety and depression, each affecting an estimated 4% of the global population (9).

Thyroid cancer (TC) is the commonest endocrine malignancy, with a global incidence of approximately 250,000 new cases in 2021 (10). TC is three times more common in females compared to males (11). Differentiated thyroid carcinoma (DTC) constitutes about 90% of all TC cases (12) and is considered an indolent tumor with good prognosis, provided adequate treatment is offered (13). Treatment for DTC typically includes total thyroidectomy and radioactive iodine (RAI) administration in selected patients. Prior to RAI treatment, TSH stimulation is considered the standard of care; this can be achieved either by levothyroxine (LT4) treatment withdrawal – rendering the patient hypothyroid, or by recombinant human TSH (rhTSH) administration – where the patient remains euthyroid (14).

There is a bidirectional relationship between poor sleep and thyroid cancer: sleep problems might be associated with increased risk of TC (15, 16), while patients with DTC have been shown to have worse sleep quality than individuals with benign thyroid disease or healthy controls (17–19). Data regarding the effects of RAI administration on sleep are scarce and controversial (17, 18), while the impact of the preparation for RAI has not been studied. The aims of the present study were to assess the sleep quality of patients with DTC undergoing RAI treatment, and to compare the effects of the type of preparation for RAI (LT4 withdrawal versus rhTSH) on sleep components.

## Materials and methods

### Study participants

This was a prospective, single-center study. One hundred and four patients programmed to receive treatment with RAI were recruited from the Endocrine Division of the University Hospital of Patras, from January to December 2023. All participants had been diagnosed with DTC (papillary or follicular thyroid cancer) and underwent total thyroidectomy with or without lymph node dissection two to eight months prior to recruitment. The need for RAI treatment and the dose administered were determined based on the 2015 American Thyroid Association Management Guidelines for Adult Patients with Thyroid Nodules and Differentiated Thyroid Cancer (14). Patients were classified into two groups, according to the type of preparation for RAI treatment: individuals in the first group had LT4 withdrawn 3–4 weeks before RAI (LT4-W group), while participants of the second group received two intramuscular doses of 0.9mg rhTSH the two days preceding RAI administration (rhTSH group). Exclusion criteria were known sleep disorders (e.g. sleep apnea, restless legs syndrome) preceding the diagnosis of DTC, severe cognitive impairment, psychiatric diseases, and alcohol or drug abuse. Participants were considered to have chronic autoimmune thyroiditis if they fulfilled at least one of the following criteria: i) positive anti-thyroid peroxidase antibodies (Anti-TPO) or anti-thyroglobulin antibodies (Anti-Tg) – the latter prior to DTC diagnosis, ii) inhomogeneous thyroid parenchyma on preoperative ultrasound (from patients’ medical records), iii) suggestive histology report after thyroidectomy. Patients were assessed in the morning of the day of RAI administration, prior to treatment. The study was conducted in accordance with the Declaration of Helsinki, and it was approved by the University Hospital of Patras Ethics Committee; all participants provided written informed consent.

### Questionnaires

#### Sleep assessment

Sleep quality was assessed with the Greek version of the Pittsburg Sleep Quality Index (PSQI). This is a widely used 19-item self-rated questionnaire assessing subjective sleep quality and quantity over a 1-month period. Seven component scores are generated from these 19 questions: subjective sleep quality, sleep latency, sleep duration, sleep efficiency, sleep disturbances, use of sleep medication, and daytime dysfunction. Each component is scored on a scale of 0 (no difficulty) to 3 (severe difficulty). The component scores are summed to produce a global score (range 0 to 21). Higher global score denotes worse sleep quality, and a cut-off of 5 is traditionally used to distinguish good (≤5) from poor (>5) sleepers (20). The PSQI has been translated in Greek, and the Greek version of the questionnaire (GR-PSQI) has been validated in a study of 209 patients (85 male and 124 female) and found to be reliable in Greek populations (21).

#### Assessment of anxiety and depression

All participants were screened for anxiety and depression using the Greek version of the Hospital Anxiety and Depression Scale (HADS). HADS is a self-assessment mood scale, designed for use in hospital departments, to identify and quantify anxiety and depression. HADS has been translated and validated in Greek, in a cohort of 120 participants (61 male and 59 female) (22).

### Hormone determinations and assays

Fasting blood samples were collected between 8 and 9a.m. on the day of RAI administration. Samples were immediately centrifuged, and the serum or plasma was collected and stored at -80°C until analysis. Serum thyrotropin (TSH), free thyroxine (FT4), thyroglobulin (Tg), anti-thyroglobulin antibodies (Anti-Tg), prolactin (PRL), cortisol, and plasma adrenocorticotropic hormone (ACTH) were measured by electrochemiluminescence immunoassays - “ECLIA” (Cobas e601, Roche Diagnostics^®^, Mannheim, Germany). The reference ranges of the kits used were 0.40−4.20 µIU/mL for TSH, 0.93−1.7 ng/dL for FT4, 3.5–77 ng/mL for Tg, <115 IU/mL for Anti-Tg, 4.04-15.20 ng/mL(men) and 4.79-23.30 ng/mL (women) for PRL, 6.2−19.4 μg/dL for cortisol, and 7.2−63.3 pg/mL for ACTH. The intra- and inter-assay precision CV (%) values were 0.7%–3.4% and 1.5%–11.2% for TSH, 0.9%-4.1% and 1.6%–5.4% for FT4, 1.4–2.4% and 1.8–3.2% for Tg, 2.0-3.9% and 3.1-7.5% for Anti-Tg, 1.3-3.0% and 1.9-5.2% for PRL, 1.0-1.7% and 1.4-2.8% for cortisol, and 0.7-2.7% and 3.7-5.4% for ACTH. The values of the lower detection limits were 0.005 μIU/mL for TSH, 0.0388 ng/dL for FT4, 0.04 ng/mL for Tg, 10.00 IU/mL for Anti-Tg, 0.047 ng/mL for PRL, 0.018 μg/dL) for cortisol, and 1.00 pg/mL for ACTH. The serum Tg assay has been calibrated against the CRM 457 international standard.

### Statistical analysis

Data was analyzed with IBM SPSS Statistics for Windows, version 28.0 (IBM Corp., Armonk, N.Y., USA). Variables were tested for normality with the Kolmogorov-Smirnov test. Categorical data are presented as number (percentage) and continuous as mean ± standard deviation (SD), regardless of their distribution. Comparisons between the two study groups were performed with the independent samples t-test for normally distributed continuous data and the Mann-Whitney U test for non-normally distributed continuous data, while the chi-squared test was used for comparisons concerning categorical variables. Adjustments for confounding variables were performed using the general linear model. Correlations were estimated by Pearson or Spearman correlation tests, as appropriate. All tests were 2-tailed and a p-value of less than 0.05 was considered significant.

## Results

One hundred and four patients (76 females and 28 males) with a mean age of 46.52 ± 14.48 years were included in our study. The mean RAI dose administered was 65.2 mCi (2,414.8 MBq) and 97 of the participants were receiving RAI for the first time.

Fifty-six individuals comprised the LT4-W group, and 48 the rhTSH group. The characteristics and hormonal parameters of the participants in each group are shown in **Table 1**. The two subgroups did not differ regarding patient gender distribution, age, histology, presence of chronic thyroiditis, RAI indication (remnant ablation, adjuvant treatment, or known structural disease), RAI dose, the percentage of patients receiving RAI for the first time, and the levels of Tg, Anti-Tg and ACTH. Patients in the rhTSH group had higher levels of TSH and FT4 and lower levels of PRL and cortisol than those in the LT4-W group.

**Table 1:** Characteristics and hormonal parameters of the participants in the two study groups, in the whole study population and in women and men separately.

Gender (F/M)	Whole study population			Women			Men		
	LT4-W (n=56)	rhTSH (n=48)	p	LT4-W (n=38)	rhTSH (n=38)	p	LT4-W (n=18)	rhTSH (n=10)	p
	38 (67.9%)/18 (32.1%)	38 (79.2%)/10 (20.8%)	0.195	38 (100%)/0	38 (100%)/0	1	0/18 (100%)	0/10 (100%)	
Age	45.34 ± 14.53	47.90 ± 14.46	0.372	42.18 ± 13.98	49.95 ± 13.58	**0.016**	52.00 ± 13.72	40.10 ± 15.77	0.062
Histology (papillary/other)	49 (87.5%)/ 7 (12.5%)	45 (93.7%)/ 3 (6.3%)	0.633	35 (92.1%)/ 3 (7.9%)	35 (92.1%)/ 3 (7.9%)	1	14 (77.7%)/ 4 (22.3%)	10 (100%)/ 0	0.265
Chronic thyroiditis (yes/no)	20 (35.7%)/ 36 (64.3%)	20 (41.7%)/ 28 (58.3%)	0.551	19 (50%)/ 19 (50%)	20 (52.6%)/ 18 (47.4%)	0.818	1 (5.6%)/ 17 (94.4%)	2 (20%)/ 8 (80%)	0.236
RAI indication (remnant ablation/adjuvant/structural disease)	15 (26.8%)/ 25 (44.6%)/ 16 (28.6%)	13 (27.1%)/ 22 (45.8%)/ 13 (27.1%)	0.985	9 (23.7%)/ 19 (50%)/ 10 (26.3%)	10 (26.3%)/ 18 (47.4%)/ 10 (26.3%)	0.961	6 (33.3%)/ 6 (33.3%)/ 6 (33.3%)/	3 (30%)/ 4 (40%)/ 3 (30%)	0.940
RAI dose	67.66 ± 30.30	62.31 ± 29.51	0.579	70.63 ± 30.15	60.61 ± 28.82	0.258	61.33 ± 30.68	71.67 ± 34.30	0.470
1^st^ time RAI	54 (96.4%)	43 (89.6%)	0.165	36 (94.7%)	34 (89.5%)	0.395	18 (100%)	9 (90%)	0.172
TSH	77.77 ± 22.93	112.88 ± 21.67	**<0.001**	80.04 ± 22.64	111.20 ± 22.13	**<0.001**	72.99 ± 23.44	119.11 ± 19.69	**<0.001**
FT4	0.11 ± 0.10	0.90 ± 0.68	**<0.001**	0.10 ± 0.11	0.93 ± 0.70	**<0.001**	0.12 ± 0.08	0.80 ± 0.65	**0.005**
Tg	11.94 ± 39.40	5.42 ± 12.15	0.098	15.59 ± 47.53	3.13 ± 5.91	0.119	4.24 ± 3.74	13.89 ± 22.63	0.944
Anti-Tg	68.80 ± 164.13	53.37 ± 132.95	0.703	75.56 ± 163.26	63.02 ± 148.71	0.865	54.52 ± 169.78	17.67 ± 9.08	0.796
PRL	20.04 ± 21.34	13.26 ± 9.12	**0.023**	23.21 ± 25.25	13.35 ± 9.59	**0.023**	13.70 ± 6.58	12.89 ± 7.56	0.769
ACTH	20.16 ± 13.38	19.49 ± 14.00	0.633	19.04 ± 12.67	17.20 ± 12.43	0.300	22.47 ± 14.85	27.72 ± 16.80	0.400
Cortisol	21.88 ± 7.67	17.24 ± 7.05	**<0.001**	20.81 ± 8.10	17.11 ± 7.02	**0.014**	23.95 ± 6.44	17.70 ± 7.53	**0.029**

LT4-W, levothyroxine withdrawal; rhTSH, recombinant human TSH. Statistically significant p values are displayed in bold.

When women were examined separately, those in the rhTSH group had a slightly higher mean age (49.95 ± 13.58 vs 42.18 ± 13.98 in the LT4-W group); the differences in hormone levels were identical to those of the whole population, and the subgroups did not differ concerning the histological characteristics or the various RAI parameters (dose, indication, first time percentage). In men, differences between the LT4-W and rhTSH groups were similar to the whole population’s, with the exception of PRL which did not differ between the subgroups (**Table 1**).

**Table 2** shows the performance of each subgroup in the sleep quality and anxiety/depression assessments (PSQI and HADS questionnaires). As shown, patients in the rhTSH group had significantly lower values in subjective quality, efficiency, daytime dysfunction, and global PSQI meaning that they had better quality and efficiency, less daytime dysfunction, and overall better sleep compared to individuals in the LT4-W group. Furthermore, there were significantly fewer poor sleepers (defined as a global PSQI score >5) in the rhTSH group (32.6% vs 53.8% in LT4-W, p=0.043 – data not shown in table). Participants in the LT4-W group had higher mean scores in the anxiety and depression components of HADS.

**Table 2:** PSQI and HADS scores in the two study groups, in the whole study population and in women and men separately.

Whole study population						
	LT4-W (n=56)	rhTSH (n=48)	p	p (partial Eta squared) – corrected for TSH, FT4, PRL, cortisol	p (partial Eta squared) – corrected for anxiety/depression	p (partial Eta squared) – corrected for TSH, FT4, PRL, cortisol and anxiety/depression
PSQI						
Subjective quality	1.22 ± 0.92	0.65 ± 0.71	**0.002**	**0.001 (0.136)**	**0.012 (0.066)**	**0.036 (0.060)**
Latency	1.29 ± 1.04	1.11 ± 1.10	0.377			
Duration	0.92 ± 1.01	0.76 ± 0.71	0.698			
Efficiency	0.72 ± 0.96	0.35 ± 0.74	**0.023**	**0.032 (0.059)**	0.282 (0.013)	0.370 (0.012)
Disturbance	1.27 ± 0.68	1.17 ± 0.66	0.443			
Sleep medication	0.29 ± 0.79	0.13 ± 0.61	0.089			
Daytime dysfunction	0.87 ± 0.82	0.52 ± 0.59	**0.030**	**0.016 (0.072)**	0.107 (0.027)	0.385 (0.011)
Global PSQI	6.60 ± 3.99	4.72 ± 3.32	**0.014**	**0.001 (0.127)**	0.162 (0.021)	0.126 (0.033)
HADS Anxiety	5.62 ± 3.85	4.32 ± 4.22	**0.046**			
HADS Depression	5.86 ± 4.22	3.55 ± 3.41	**0.005**			
Women						
	LT4-W (n=38)	rhTSH (n=38)	p	p (partial Eta squared) – corrected for TSH, FT4, PRL, cortisol	p (partial Eta squared) – corrected for anxiety/depression	p (partial Eta squared) – corrected for TSH, FT4, PRL, cortisol and anxiety/depression
PSQI						
Subjective quality	1.24 ± 0.91	0.76 ± 0.71	**0.023**	**0.014 (0.109)**	**0.023 (0.071)**	0.090 (0.058)
Latency	1.46 ± 1.07	1.18 ± 1.11	0.276			
Duration	0.91 ± 1.01	0.82 ± 0.73	0.953			
Efficiency	0.80 ± 1.02	0.42 ± 0.79	**0.050**	0.192 (0.033)	0.107 (0.038)	0.426 (0.014)
Disturbance	1.42 ± 0.68	1.18 ± 0.69	0.143			
Sleep medication	0.24 ± 0.68	0.16 ± 0.68	0.272			
Daytime dysfunction	1.03 ± 0.85	0.58 ± 0.60	**0.018**	**0.006 (0.133)**	**0.033 (0.064)**	**0.038 (0.085)**
Global PSQI	7.14 ± 4.11	5.16 ± 3.40	**0.029**	**0.025 (0.094)**	0.051 (0.060)	0.136 (0.047)
HADS Anxiety	6.00 ± 4.02	5.16 ± 4.34	0.293			
HADS Depression	5.78 ± 4.06	4.11 ± 3.57	0.063			
Men						
	LT4-W (n=18)	rhTSH (n=10)	p	p (partial Eta squared) – corrected for TSH, FT4, PRL, cortisol	p (partial Eta squared) – corrected for anxiety/depression	p (partial Eta squared) – corrected for TSH, FT4, PRL, cortisol and anxiety/depression
PSQI						
Subjective quality	1.18 ± 0.95	0.13 ± 0.35	**0.006**	0.074 (0.159)	0.232 (0.067)	0.296 (0.064)
Latency	0.48 ± 0.90	0.75 ± 1.04	0.628			
Duration	0.94 ± 1.03	0.50 ± 0.53	0.440			
Efficiency	0.53 ± 0.80	0.00 ± 0.00	0.175			
Disturbance	0.94 ± 0.56	1.10 ± 0.57	0.570			
Sleep medication	0.41 ± 1.00	0.00 ± 0.00	0.473			
Daytime dysfunction	0.53 ± 0.62	0.25 ± 0.46	0.374			
Global PSQI	5.47 ± 3.57	2.63 ± 2.00	**0.018**	0.163 (0.100)	0.923 (0.000)	0.768 (0.005)
HADS Anxiety	4.83 ± 3.43	1.20 ± 1.40	**0.004**			
HADS Depression	6.00 ± 4.66	1.50 ± 1.51	**0.001**			

PSQI scores are displayed both unadjusted and corrected for hormonal parameters, anxiety/depression, and both. Statistically significant p values are displayed in bold.

Women in the rhTSH group showed better quality and efficiency, less daytime dysfunction, and overall better sleep than their LT4-W counterparts, while in men the rhTSH group had better scores in the quality and global components of the PSQI (**Table 2**). Poor sleepers were less in the rhTSH group in both women (36.8%) and men (12.5%) compared to the LT4-W group (57.1% and 47.1%, respectively), but the statistical significance was marginal (p=0.082 and 0.093, respectively). Men subjected to LT4-W had higher scores in HADS anxiety and depression compared to those receiving rhTSH; in women there was a trend for higher depression in the LT4-W group (**Table 2**).

Since there were significant differences between the groups in hormonal parameters and in anxiety/depression levels, we further analyzed our data regarding sleep quality after correction for these parameters. Participants in the rhTSH group continued to demonstrate significantly lower scores in the same PSQI domains after correction for TSH, FT4, PRL, and cortisol levels. However, when corrected for HADS scores or for HADS scores and hormonal parameters, the only significant difference remaining between the groups was in the domain of subjective quality. Notably, the effect size of preparation type in the adjusted models created was medium to large, as estimated by the Partial Eta Squared (**Table 2**).

In women the same motif was observed after correcting the PSQI scores for hormonal and anxiety/depression levels, with the exception that the difference between the groups in daytime dysfunction persisted after all corrections. In men all initial differences disappeared after correction for hormonal and/or HADS levels.

All PSQI domains showed significant correlation with anxiety levels, while depression levels were significantly correlated with all PSQI components except duration; higher levels of anxiety or depression, were correlated with higher PSQI scores (worse sleep). The correlations were mostly of moderate strength (**Table 3**). The only significant correlation between PSQI components and hormones was a weak negative correlation of TSH with subjective quality (Spearman’s rho=-0.216, p=0.031). Finally, depression was negatively associated with FT4 (Spearman’s rho=-0.229, p=0.030), and positively with PRL (Spearman’s rho=0.237, p=0.018) levels.

**Table 3:** Spearman correlations between PSQI domains and anxiety/depression (HADS).

PSQI domainsc	HADS anxiety	HADS depression
Subjective quality	r=0.477, p<0.001	r=0.461, p<0.001
Latency	r=0.282, p=0.005	r=0.294, p=0.004
Duration	r=0.310, p=0.002	r=0.183, p=0.075
Efficiency	r=0.340, p=0.001	r=0.335, p=0.001
Disturbance	r=0.438, p<0.001	r=0.361, p<0.001
Sleep medication	r=0.215, p=0.031	r=0.387, p<0.001
Daytime dysfunction	r=0.527, p<0.001	r=0.477, p<0.001
Global PSQI	r=0.584, p<0.001	r=0.554, p<0.001

## Discussion

Our study showed that, in patients with DTC undergoing RAI treatment, the type of preparation for RAI affects sleep; individuals prepared with LT4 withdrawal had significantly worse sleep quality compared to those prepared with rhTSH. This difference did not seem to be mediated by discrepancies in hormonal levels between the groups, but rather by increased anxiety and depression in the LT4-W subjects.

Sleep disturbance is a common and impactful comorbidity among patients with cancer, with prevalence rates at least double those observed in the general population (23, 24). Sleep problems have significant negative effects on the patients’ quality of life, and poor sleep has been correlated with adverse health outcomes such as immune suppression, cognitive deficits, cardiovascular disease, insulin resistance and type 2 diabetes (25, 26). There are many factors, (physiological, psychological and/or treatment-related) contributing to the impaired sleep quality of cancer patients, but the most important seems to be fear; fear of the diagnosis itself, of treatment(s), complications, and of recurrence (27, 28).

Sleep problems are also common in thyroid cancer, and in particular in DTC survivors. Studies have shown that sleep quality tends to be poorer in such individuals compared to healthy controls but also compared to patients with benign thyroid nodules undergoing thyroid surgery (18, 19). It seems, however, that the DTC status might be a confounding factor, as the latter studies included patients with active disease, whereas Teliti et al. found no difference in the rates of insomnia and poor sleep between disease-free individuals with DTC and subjects who had undergone surgery for benign thyroid pathology (29). Moreover, among patients with active thyroid cancer, metastatic disease is associated with worse sleep parameters (18). In a longitudinal study Koo et al. showed that in DTC patients sleep disturbance peaks prior to surgery and gradually diminishes over subsequent months, returning to levels comparable to the general population after several years (17). Regarding the effect of treatment with RAI, this study demonstrated no harmful effect on sleep quality (17), while He et al. found that PSQI scores and the proportion of poor sleepers among patients with DTC increased after RAI administration (18). Our study confirmed the increased prevalence of disturbed sleep in DTC patients (our cohort had an overall 44% of poor sleepers, compared to 15.9-41% in the general population (30, 31)). The difference in sleep parameters between our two study subgroups cannot be explained by differences in DTC status; the groups included individuals with similar histologic tumor characteristics, similar rates of the 3 main indications for RAI treatment, and similar mean RAI dose administered, meaning that disease severity was comparable between the groups. All patients were evaluated on the day of RAI administration, so they were equally affected by RAI treatment-related stress, and they were not influenced by the results of the post-treatment whole body scan. Notably, sleep difficulty is also considered a putative factor predisposing to TC (15) via circadian clock dysregulation and other mechanisms (32). Unfortunately, we do not have data regarding the sleep characteristics of our patients before DTC diagnosis to confirm or reject chronic sleep problems in our population.

There is a close, reciprocal relationship between sleep and thyroid function. Sleep is a significant contributor in the normal physiology of the hypothalamus-pituitary-thyroid (HPT) axis. Thyroid hormone secretion follows a circadian rhythm, with TSH levels peaking at night, while free T3 and T4 levels display less consistent patterns. Sleep decreases the nocturnal TSH rise and thyroid hormone levels, while sleep deprivation has been associated with alterations in TSH and thyroid hormones, which are dependent on the duration of sleep restriction and on gender (1, 4, 5). Nazem et al. showed that, among young adults, poor sleepers had higher TSH and FT4 levels than good sleepers (33). However, a recent Mendelian randomization study did not find indication of causal links between thyroid function and genetically predicted sleep characteristics (34). On the other hand, primary thyroid dysfunction is also associated with sleep problems. Sleep disruption with increased latency, difficulty maintaining sleep and decreased sleep duration and efficiency are very common in hyperthyroidism (1, 6). Hypothyroidism also adversely affects sleep; untreated overtly hypothyroid patients have disrupted sleep architecture characterized by decreased deep and rapid-eye-movement sleep relative to healthy controls (35). Subclinical hypothyroidism has been positively correlated with decreased sleep quality and/or duration in the majority of relevant studies (36). With regard to the possible mechanisms linking hypothyroidism and sleep dysfunction, one possibility might be that symptoms of thyroid hormone deficiency such as arthralgias/myalgias, cold intolerance and anxiety could exacerbate sleeping difficulties (6). Hypothyroid patients may also have a higher prevalence of comorbidities, which increases the risk of insomnia (7). Hypothyroidism may indirectly affect sleep by triggering or exacerbating obstructive sleep apnea (OSA); indeed, the prevalence and severity of OSA is higher in hypothyroid subjects compared to the general population (37). Finally, disrupted connectivity between the suprachiasmatic nucleus and other central nervous system structures in hypothyroid patients has been proposed to affect normal circadian functions and sleep (38). Thyroid autoimmunity is the most common cause of hypothyroidism. In most studies, thyroid antibody positivity did not seem to affect the relationship between poor sleep quality and elevated TSH (36). Our study included two groups of patients: one with severe, short-standing (less than 1 month’s duration) hypothyroidism (LT4-W), and one with normal thyroid hormone levels (rhTSH). The two groups had significant differences in several sleep indices as measured by the PSQI, both in the whole study population and in women; however, the worse sleep quality of the LT4-W group could not be attributed to the difference in FT4 and TSH, as statistical correction for hormonal levels did not attenuate the between-groups difference in PSQI components. In men, the two subgroups differed in only two PSQI components (subjective quality and global score), and there was marginal significance between groups after correction for hormonal levels, possibly due to the small number of male patients. The lack of significant associations between thyroid hormone levels and PSQI scores seems counter-intuitive, given the well-established correlation of chronic hypothyroidism with poor sleep. However, it is important to note that our study included only patients with short-term hypothyroidism, and the duration of hypothyroidism may play a significant role in the relationship between thyroid dysfunction and sleep. Moreover, the levels of FT4 in the rhTSH group (“euthyroid” subjects) were in the low-normal range, and this might have limited the effect of FT4 in the performed statistical analyses.

The patients in the LT4-W subgroup of our study had significantly higher levels of anxiety and depression compared to the individuals comprising the rhTSH group. Increased anxiety/depression seemed to be the main factor explaining the worse sleep experienced by subjects of the LT4-W group, as the between-groups differences in multiple PSQI components were lost after correction for anxiety/depression levels. Analysis by gender revealed that men in the LT4-W group were more anxious/depressed than their rhTSH counterparts, and that their impaired sleep quality was more dependent on these psychological than on hormonal variables (statistical correction of the PSQI components for hormonal parameters led to marginally significant differences between the subgroups, while correction for HADS scores completely abolished the differences). In women, the subgroups had similar levels of anxiety and the rhTSH group had a trend for higher depression; here, correction of the PSQI indices for psychological parameters had a lesser impact than in men or the whole population. The relationship between depression and sleep quality is strong and well-established. Depression causes multiple sleep problems, the most common being insomnia, and changes in sleep are among the diagnostic criteria for depression (39, 40). Sleep disturbance and poor sleep quality lead to exacerbation of depressive symptoms and might increase suicide risk, while sleep improvement may improve the outcomes in depressive patients (41). Likewise, increased anxiety/stress is also strongly correlated with impaired sleep quality (42, 43). Studies have also revealed that the relationship between anxiety/depression and sleep disorders is bidirectional, meaning that each contributes to the development but is also a consequence of the other (41). It is not easy to explain why our patients in the LT4-W group were more anxious and/or depressed than those in the rhTSH group. As already mentioned, the two groups had similar disease severity, and it is anticipated that they were equally affected by any potential stress related to RAI administration. Lower FT4 levels in the LT4-W patients may have a role; hypothyroidism, both overt and subclinical, has been positively correlated with the risk of depression, although the association seems to be modest (44, 45). This was also reflected in our data by a weak negative correlation between FT4 and depression. However, the established association of hypothyroidism with depression pertains to chronic hypothyroidism and our LT4-W patients suffered from short-term hypothyroidism; hence, attributing their increased anxiety/depression to low FT4 levels might not be sufficiently justified based on the existing literature. Chronic autoimmune thyroiditis, even without overt hypothyroidism, has been associated with higher rates of anxiety/depression, with possible implications on sleep quality. There was no significant difference regarding the presence of chronic thyroiditis in the two subgroups of our study. Notably, the diagnosis of thyroiditis was not based solely on the presence of positive thyroid autoantibodies; histology reports of thyroidectomies were also reviewed, as a significant subset of hypothyroid patients have antibody-negative thyroiditis (46). Patients in the LT4-W group had also higher cortisol and prolactin levels than their counterparts of the rhTSH group. There is a bidirectional relationship between cortisol and depression, meaning that elevated cortisol can increase vulnerability to depression, while depression can cause increased cortisol levels (47, 48). A similar model has been observed with prolactin: its levels can be increased by any type of stress, whereas hyperprolactinemia in patients with prolactinomas has been linked with several psychological symptoms, such as anxiety and depression (49, 50). It is possible that the higher cortisol and prolactin levels in the LT4-W subjects are either a cause or a consequence of increased anxiety or depression, even though the differences between the groups -though statistically significant- were of small magnitude and the group means within the normal or high-normal range. Lastly, we cannot exclude the possibility that other factors, such as the anticipation of imminent hypothyroidism in the LT4-W subjects or mere chance being responsible for the observed difference in anxiety/depression levels. Notably, even after controlling for all hormonal and psychological variables, LT4-W patients continued to report impaired subjective sleep quality and additionally women continued to present daytime dysfunction. These findings suggest that additional, unidentified factors influence participants’ sleep quality, especially in females.

Our study has several limitations. It was a cross-sectional study, that lacks evidence regarding the hormonal status and sleep parameters of the participants before the diagnosis of cancer, before the selection of the type of preparation for RAI treatment, and after RAI administration. Moreover, we did not collect data concerning the overall quality of life of the patients, which is a crucial modifier of sleep quality.

In conclusion, our study revealed that in patients with DTC receiving RAI, preparation with rhTSH may be associated with better sleep quality compared to LT4 withdrawal. Increased depression and anxiety seem to be the driving force behind the sleep impairment observed in LT4-W subjects. Further research is warranted to confirm these findings, identify more factors (hormonal, psychological, etc.) affecting the sleep quality of patients undergoing RAI treatment, and develop strategies to support improved well-being and sleep quality in these patients.

## Data Availability

The raw data supporting the conclusions of this article will be made available by the authors, without undue reservation.
